# In This Issue

**DOI:** 10.1002/cfg.409

**Published:** 2004-06


**Comparative and Functional Genomics**
*Comp Funct Genom* 2004; **5**: 303.
Published online in Wiley InterScience (www.interscience.wiley.com). **DOI:** 10.1002/cfg.409

### In this issue

### Genomic assessment of novel antibacterial targets

**Tom White** and **Douglas Kell** have used a computational approach to identify novel targets for antibacterial drugs. They used a range of criteria, such as sequence conservation across a range of pathogenic bacteria, and absence of a human homologue, to score and rank bacterial genes for their potential as drug targets.

### Evolution and cellular function of monothiol glutaredoxins

**Felipe Vilella** and colleagues used phylogenetic profiling to predict that bacterial monothiol glutaredoxins participate in the iron–sulphur cluster (ISC) assembly machinery. Their computational study predicted a functional interaction between these proteins, which was confirmed by two-hybrid analyses.

### GFP fusions for subcellular localizations using reverse transfection microarrays

**Ella Palmer** and **Tom Freeman** present the results of an investigation into the use of N- and C-terminal GFP fusion constructs with reverse transfection microarrays to determine subcellular localizations of proteins.

### Mapping GO into UMLS

**Jane Lomax** and **Alexa McCray** describe their work in mapping the Gene Ontology (GO) into the National Library of Medicine's Unified Medical Language System (UMLS). This work highlighted issues in both terminology systems and should contribute to linking the language and practices of clinical medicine to those of genomics.

### Developing the *Arabidopsis* information resource

The *Arabidopsis* Information Resource (TAIR) is a web-based community database for the model plant *Arabidopsis thaliana*. In this review, **Danforth Weems *et al.*** describe how TAIR was built and is being maintained.

### Conference calendar

A listing of genomics-related conferences planned for October–December 2004.

Copyright © 2004 John Wiley & Sons, Ltd.

